# Medicinal Plants of the Australian Aboriginal Dharawal People Exhibiting Anti-Inflammatory Activity

**DOI:** 10.1155/2016/2935403

**Published:** 2016-12-27

**Authors:** Most A. Akhtar, Ritesh Raju, Karren D. Beattie, Frances Bodkin, Gerald Münch

**Affiliations:** ^1^Department of Pharmacology, School of Medicine, Western Sydney University, Sydney, NSW, Australia; ^2^Department of Pharmacy, University of Rajshahi, Rajshahi, Bangladesh; ^3^National Institute of Complementary Medicine, Western Sydney University, Sydney, NSW, Australia

## Abstract

Chronic inflammation contributes to multiple ageing-related musculoskeletal and neurodegenerative diseases, cardiovascular diseases, asthma, rheumatoid arthritis, and inflammatory bowel disease. More recently, chronic neuroinflammation has been attributed to Parkinson's and Alzheimer's disease and autism-spectrum and obsessive-compulsive disorders. To date, pharmacotherapy of inflammatory conditions is based mainly on nonsteroidal anti-inflammatory drugs which in contrast to cytokine-suppressive anti-inflammatory drugs do not influence the production of cytokines such as tumour necrosis factor-*α* or nitric oxide. However, their prolonged use can cause gastrointestinal toxicity and promote adverse events such as high blood pressure, congestive heart failure, and thrombosis. Hence, there is a critical need to develop novel and safer nonsteroidal anti-inflammatory drugs possessing alternate mechanism of action. In this study, plants used by the Dharawal Aboriginal people in Australia for the treatment of inflammatory conditions, for example, asthma, arthritis, rheumatism, fever, oedema, eye inflammation, and inflammation of bladder and related inflammatory diseases, were evaluated for their anti-inflammatory activity in vitro. Ethanolic extracts from 17* Eucalyptus* spp. (Myrtaceae) were assessed for their capacity to inhibit nitric oxide and tumor necrosis factor-*α* production in RAW 264.7 macrophages.* Eucalyptus benthamii* showed the most potent nitric oxide inhibitory effect (IC_50_  5.57 ± 1.4 *µ*g/mL), whilst* E. bosistoana, E. botryoides, E. saligna, E. smithii, E. umbra, and E. viminalis *exhibited nitric oxide inhibition values between 7.58 and 19.77 *µ*g/mL.

## 1. Introduction

Inflammation is an important biological process and is essential to maintain the body's homeostasis, to fight against pathogens effectively, and to repair the damaged tissue [1]. However when uncontrolled and chronic, inflammation gives rise to a number of (often age related) diseases including asthma, rheumatoid arthritis, inflammatory bowel disease, Crohn's disease, and tendonitis. Furthermore, a chronic inflammatory response with accompanying oxidative stress is a significant force driving the progression of peripheral diseases like atherosclerosis, diabetes, and metabolic syndrome, as well as neurodegenerative diseases such as multiple sclerosis, Parkinson's disease, and Alzheimer's disease [2–5].

While some chronic/remitting neurological diseases, such as multiple sclerosis, have long been recognized as inflammatory, the term “neuroinflammation” is now applied to chronic activation of microglia and astroglia that do not reproduce the classic characteristics of inflammation in the periphery but may cause neurodegeneration [6–8]. Some examples of diseases characterized by neuroinflammation are Alzheimer's disease (AD) and Parkinson's disease and even autism-spectrum and obsessive-compulsive disorders [9–12]. Microglial and astroglial activation, accompanied by increased levels of proinflammatory mediators such as TNF-*α*, IL-1*β* and IL-6, prostaglandins, and reactive oxygen and nitrogen species, as well as reactive carbonyl species and advanced glycation end products, is observed in the AD brain at all stages of the disease [13–18]. Genetic and pharmacoepidemiological studies also point to the importance of inflammation in AD. For example, three immune-relevant genes were shown to be associated with an increased risk of AD; these are CLU (clusterin), CR1 (complement receptor 1), and TREM2 (triggering receptor expressed on myeloid cells 2) [19].

Consequently, targeting chronic neuroinflammation, for example, with plant-derived anti-inflammatory compounds, has been suggested as a promising disease-modifying treatment for many neurodegenerative diseases including AD [12, 20–27].

At present, both steroidal and nonsteroidal anti-inflammatory drugs (NSAIDs) are used to treat inflammation. NSAIDs in particular can cause severe side effects, most importantly gastric ulcers. NSAIDs are specifically designed as inhibitors of cyclooxygenase (COX) enzymes and, in contrast to CSAIDs, do not influence the production of proinflammatory cytokines such as TNF-*α* or free radicals such as nitric oxide [28]. CSAIDs specifically target p38 MAPK and NF-*κ*B signalling pathways to inhibit cytokine-mediated events with demonstrated efficacy in a range of animal models [29, 30].

Activated inflammatory cells produce a variety of chemokines and cytokines, reactive oxygen species (ROS), reactive nitrogen species (RNS), free radicals, and prostaglandins [31–33] and cease to produce neuroprotective factors such as glutathione [6, 7].

Excessive production of inflammatory cytokines and reactive radical species can damage cellular biomolecules like proteins, lipids, and carbohydrates as well as nucleic acids, leading to cellular and tissue damage, which further perpetuates the inflammatory cascade. Therefore, pharmacological compounds with the ability to attenuate the production of these inflammatory molecules may have potential for the treatment of many inflammatory diseases including AD [21, 22, 28, 34, 35].

The use of natural substances, especially those derived from plants, in order to prevent, manage, or cure diseases is a centuries-old practice which has led to the discovery of many modern pharmaceuticals. In recent years, the search for novel anti-inflammatory drugs from a wide range of medicinal plant resources has been intensified, and a variety of plant secondary metabolites including apigenin, curcumin, cinnamaldehyde, and resveratrol have already been found to suppress inflammatory responses [21, 22, 28].

For example, turmeric (*Curcuma longa*) and its main ingredient curcumin, which has long been used for treatment of rheumatic disorders, exerts both anti-inflammatory and antiatherosclerotic effects [23, 36]. Ginger extract (*Zingiber zerumbet*) and its main active compound, 3-*O*-methyl kaempferol, significantly attenuated carrageenan-induced mouse paw oedema in an in vivo model and were also found to inhibit the production of nitric oxide (NO) and prostaglandin E_2_ (PGE_2_), as well as iNOS expression in a cell culture model. Aqueous and hydroalcoholic as well as ethanolic extracts from another ginger species (*Zingiber officinale*) demonstrated significant anti-inflammatory activity and its active constituent [6] gingerol again showed anti-inflammatory activity by inhibiting the production of NO and PGE_2_ [37] and was also successful in inhibiting carrageenan-induced rat paw oedema [38].

Triterpenoid saponins, from the Australian desert tree* Acacia victoriae,* have shown anti-inflammatory effects* via* inhibiting activation of NF-*κ*B, by preventing its nuclear localization and inhibiting its ability to bind to DNA [39]. Another Australian indigenous plant* Tinospora smilacina* is claimed to possess long chain unsaturated fatty acids which possess anti-inflammatory properties [40]. The fruits of the Australian native Kakadu plum (*Terminalia ferdinandiana*), Illawarra plum (*Podocarpus elatus*), and Native currant (*Acrotriche depressa*) also exhibited significant anti-inflammatory activity [41].

There is large scope to investigate Australian native plants for their bioactivity and chemical constituents [42]. Traditional medicine is still practised by the many tribal Aboriginal people, particularly in Central and Northern Australia and this ethnomedicinal knowledge is recorded in some cases [43]. The “Dharawal Pharmacopeia” written by botanist and Aboriginal Elder Frances Bodkin (known as Aunty Fran) is a compilation of the Aboriginal medicinal and ceremonial uses (and corresponding taxonomic identification) of thousands of native Australian plants. Of interest to our research, a number of plant species described in the Dharawal pharmacopeia have been claimed to possess anti-inflammatory activities (Table 1) [44, 45]. Plants from* Eucalyptus* species have special importance for the Dharawal indigenous people and are used for their anti-inflammatory activity along with other medicinal uses as well as for shelter and weapons. As stated in the Dharawal pharmacopeia, Eucalypts are mostly distributed in Blue Mountains, Southern Highlands, Woronora Plateau, and coastal area of New South Wales, Australia.

The aim of our research is to evaluate the anti-inflammatory activity of Australian native plants with ethnopharmacological importance and subsequently characterise the bioactive components. In this manuscript, dried extracts from 17* Eucalyptus* spp. were evaluated for anti-inflammatory activity* via* the suppression of NO and TNF-*α* production induced by lipopolysaccharide (LPS) and interferon gamma (IFN-*γ*) in RAW 264.7 cells. Cytotoxicity of the crude extracts was also examined using an Alamar blue cell viability assay.

## 2. Materials and Methods

### 2.1. Plant Material

Plants known to be used by the Dharawal people (also known as Tharawal) to treat inflammation and related illnesses were selected under the guidance of botanist and Aboriginal Elder Auntie Fran (Frances Bodkin) and the Dharawal pharmacopeia. Leaf material of 17* Eucalyptus* spp. was collected in the month of August, 2015 from the “Australian Botanic Gardens” at Mount Annan, NSW, Australia (Table 1).

### 2.2. Chemicals and Reagents

Ethanol was purchased from Chem-Supply (Gillman, SA, Australia); bovine serum albumin, lipopolysaccharide (*E. coli* serotype-0127:B8), EDTA,* N*-(1-napthyl) ethylenediamine dihydrochloride, benzylpenicillin G sodium salt, resazurin sodium salt (10%), streptomycin, sulphanilamide, 3,3′,5,5′-tetramethylbenzidine (TMB), trypan blue, and Dulbecco's Modified Eagle's Medium (DMEM) were purchased from Sigma-Aldrich (Castle Hill, NSW, Australia). GIBCO, fetal bovine serum (FBS), and glutamine were purchased from Life Technologies (Mulgrave, VIC, Australia). Murine interferon-*γ* (IFN-*γ*) and TNF-*α* ELISA kits were purchased from PeproTech Asia (Rehovot, Israel). Citric acid and monosodium dihydrogen carbonate (NaH_2_CO_3_) were from AJAX Chemicals (Auburn, NSW, Australia). Tween-20 was from Amresco (Solon, Ohio, USA). Methanol, monosodium phosphate (NaH_2_PO_4_), disodium phosphate (Na_2_HPO_4_), sodium chloride (NaCl), and sulfuric acid (H_2_SO_4_) were from Merck (Darmstadt, Germany). Sodium carbonate (Na_2_CO_3_) was BDH brand supplied by Merck Pty. Ltd. (Kilsyth, VIC, Australia).

### 2.3. Extraction of Plants Leaves for Biological Assays and HPLC and MS Analysis

Approximately 40 g of fresh leaf material from each plant was extracted using absolute ethanol. The leaves were first cut into small pieces with scissors and then ground to a coarse powder using a hand blender. The coarse powder was filled into the thimbles of an accelerated solvent extraction system (Buchi B-811, Switzerland) and then extracted under standard soxhlet mode (for 2 × 15 minutes cycles). The volume of the extracts was reduced to ca. 2–4 mL using a rotary evaporator and then evaporated to dryness with nitrogen gas for biological assays. Percentage yields (g/g% fresh weight) are recorded in Table 2.

### 2.4. Maintenance and Preparation of RAW 264.7 Macrophages

RAW 264.7 macrophages were grown in 175 cm^2^ culture flasks on DMEM (Dulbecco's Modified Eagle's Medium) containing 5% FBS (fetal bovine serum) that was supplemented with antibiotics (1%) and glutamine (1%). The cell line was maintained in 5% CO_2_ at 37°C, with media being replaced every 3-4 days. Once cells had grown to confluence in the culture flask, they were removed using a rubber policeman cell scraper, as opposed to using trypsin, which can remove membrane-bound receptors such as RAGE. The cell suspension was concentrated by centrifugation for 3 min at 900 rpm and resuspended in a small volume of fresh DMEM (with 1% antibiotics and 5% FBS). Cell densities were estimated using a Neubauer counting chamber. Cell concentration was adjusted with DMEM (with 1% antibiotics and 5% FBS) to obtain 60000 cells/100 *µ*L cell suspension. The 100 *μ*L cell suspension was then dispensed into the inner wells of 96-well plates. Plates were incubated at 37°C and 5% CO_2_ for 18 h before the activation experiments were carried out.

### 2.5. Activation of RAW 264.7 Macrophages

From each well, the media were removed and replaced with fresh DMEM containing 0.1% FBS. For assays with extracts, a 90 *μ*L volume of the dilutions in DMEM (with 0.1% FBS) was added an hour prior to addition of the activator. Due to the often inconsistent nature of LPS at activating cells, a combination of LPS (10 *μ*g/mL) and IFN-*γ* (10 U/mL), both in DMEM (with 0.1% FBS), was used for activation. A maximum dose of the extracts used is 900 *µ*g/mL and diluted serially by 50% up to a minimum of 10 doses (900, 450, 225, 112.5, 56.25, 28.125, 14.062, 7.031, 3.515, 1.7578, and 0.8789 *µ*g/mL in the wells, resp.). After activation, the cells were incubated for 24 h at 37°C and 5% CO_2_ and then NO and TNF-*α* inhibition and cell viability were determined. Cells with media alone were used as negative control and activated cells used as positive control.

### 2.6. Determination of Nitric Oxide Production by Griess Assay

Nitric oxide was determined by Griess reagent quantification of nitrite, one of its stable reaction products. Griess reagent was freshly made up of equal volumes of 1% sulfanilamide and 0.1% naphthylethylene-diamine in 5% HCl. In the presence of nitrite this reagent forms a violet colour. From each well, 50 *μ*L of supernatant was transferred to a fresh 96-well plate and mixed with 50 *μ*L of Griess reagent, and the colour produced was measured at 540 nm in a microplate reader (Bio-Rad, Australia). The remaining supernatant from each well was used for a TNF-*α* assay using commercial sandwich ELISA development kits (catalog number: 900-K54; PeproTech, USA).

### 2.7. Determination of Cell Viability by Alamar Blue Assay

The Alamar Blue assay is a colorimetric assay involving the cellular reduction of resazurin to resorufin. Alamar Blue solution [100 *µ*L of 10% Alamar Blue (resazurin) in DMEM medium] was added to each well and incubated at 37°C for 1-2 h. After incubation, fluorescence was measured (excitation at 530 nm and emission at 590 nm) using a POLARstar Omega microplate reader (BMG Labtech, Mornington, Australia) and expressed as a percentage of that in control wells after background fluorescence was subtracted.

### 2.8. TNF-*α* Determination by ELISA

The supernatants obtained from each well (remaining supernatant after 24 hours of activation) were diluted 30 times using diluent (0.1% w/v bovine serum albumin and 0.05% v/v tween-20 in PBS [1.9 mM NaH_2_PO_4_, 8.1 mM Na_2_HPO_4_, and 154 mM NaCl; pH 7.4]) and were used for determination of TNF-*α* using a commercial sandwich ELISA (catalog number: 900-K54; Peprotech, USA) according to the manufacturer's protocol. Capture antibody was used at a concentration of 1.25 *μ*g/mL in PBS. To make a standard curve TNF-*α* (10 ng/mL standard) was diluted serially by 50% up to a minimum of 10 doses (10, 5, 2.5, 1.25, 0.625, 0.312, 0.156, 0.078, 0.039, 0.019, and 0.0097 ng/mL in the wells, resp.) and was used as the internal standard. TNF-*α* was detected with a biotinylated second antibody and an avidin peroxidase conjugate with TMB as detection reagent. After ~30 min, the reaction was stopped using 0.5 M sulfuric acid, and the absorbance was measured at 450 nm of measurement filter with a 655 nm of reference filter. The absorbance data was expressed as a percentage of that in control wells after conversion of the concentrations by using a standard curve constructed with defined concentrations of TNF-*α*. Curve fitting of this standard curve and extrapolation of experimental data were performed using nonlinear regression analysis.

### 2.9. Data Presentation and Analysis

As the experiments were done in triplicates, the results were expressed as the mean ± SEM. In addition, linear relationships and significance tests of these data sets were also conducted. GraphPad Prism version 6.01 (GraphPad Software Incorporated, USA) was used for growth curve analysis in dose-dependent experiments and to determine the IC_50_ values for NO and TNF-*α* inhibition as well as LC_50_.

## 3. Results and Discussion

In this study, leaves from 17 different* Eucalyptus *spp. were collected in the month of August, 2015. Approximately 40 g of leaves from each of* Eucalyptus acmenoides, E. benthamii, E. bosistoana, E. botryoides, E. eximia, E. globoidea, E. gummifera, E. maculate, E. notabilis, E. paniculata, E. pilularis, E. punctate, E. resinifera, E. saligna, E. smithii, E. umbra, and E. viminalis* were extracted using absolute ethanol (Table 2).

The RAW 264.7 murine macrophages release NO and TNF-*α* when exposed to bacterial LPS and IFN-*γ* and on this principle, has become an established experimental model to evaluate in vitro anti-inflammatory activity of extracts [28]. For the purpose of interpretation, the IC_50_ values of NO inhibition are divided into three groups: extracts with IC_50_ < 20 *µ*g/mL are considered as highly potent extracts; a value between 21 and 80 *µ*g/mL is considered as moderately potent, and an IC_50_ < 80 *µ*g/mL is considered as an extract with low potency.

The highest concentration of ethanolic crude extract tested in the anti-inflammatory assay was 900 *µ*g/mL with 0.5-fold serial dilutions.* Eucalyptus benthamii, E. bosistoana, E. botryoides, E. saligna, E. smithii, E. umbra, and E. viminalis *leaf extracts showed the highest activity for NO inhibition with IC_50_ values of 5.57, 7.58, 16.65, 19.77, 17.62, 17.69, and 8.0 *µ*g/mL, respectively (Table 3, Suppl. Figure 1). The extracts from* Eucalyptus acmenoides, E. eximia, E. notabilis,* and* E. pilularis* showed moderate inhibition of NO with IC_50_ values of 56.93, 34.14, 53.84, and 76.17 *µ*g/mL, respectively. Six other species,* E. globoidea, E. gummifera*,* E. maculata*,* E. paniculata, E. punctata, and E. resinifera*, presented low inhibition of NO with IC_50_ values of 82.9, 108.17, 99.94, 130.7, 120.4, and 81.21 *µ*g/mL, respectively (Suppl. Figure 1).

The plant extracts also showed promising TNF-*α* inhibitory activity (Table 3) with IC_50_ values of 2.06, 8.53, 19.02, 3.41, 2.41, 10.2, and 16.68 *μ*g/mL for* E. benthamii, E. bosistoana, E. botryoides, E. saligna, E. smithii, E. umbra, and E. viminalis*, respectively, which are the same plants in our highly potent NO inhibitor group. On the other hand, the moderately potent extracts from* E. acmenoides, E. eximia, E. notabilis, and E pilularis *showed TNF-*α* IC_50_ values of 16.53, 4.82, 27.48, and 21.09 *μ*g/mL, respectively (Suppl. Figure 1), whereas extracts from* E. globoidea, E. gummifera, E. maculata, E. paniculata, E. punctata, and E. resinifera* exhibited comparatively lower inhibition of TNF-*α* production with IC_50_ values of 50.73, 82.73, 136.34, 334.86, 115.73, and 62.11 *μ*g/mL, respectively, which are the plants in our low potency group (Suppl. Figure 1).

The use of Alamar Blue (resazurin) to measure cytotoxicity is an established technique [46]. The results of cytotoxicity (LD_50_) of our leaf extracts are shown in Table 3. The plants of our highly potent group were also relatively toxic with LC_50_ values of 22.34, 37.17, 108.40, 101.01, 38.96, 236.5, and 31.92 for* E. benthamii, E. bosistoana, E. botryoides, E. saligna, E. smithii, E. umbra*, and* E. viminalis*, respectively, whereas, plants of the lower potency group showed lower toxicity with higher LD_50_ values of 464.74, 313.45, 540.46, 268.59, 522.84, and 268.59 for* E. globoidea, E. gummifera*,* E. maculata*,* E. paniculata, E. punctata*,* and E. resinifera*, respectively. Plants with moderate potency showed a wide range of cytotoxicity with LD_50_ values of 296.22, 64.14, 332.44, and 374.74 for* E. acmenoides, E. eximia, E. notabilis, *and* E. pilularis*, respectively (Suppl. Figure 1).

In future experiments, we will purify the most potent extracts to identify the most active compounds. One major candidate for carrying the anti-inflammatory activity could be 1,8- cineole, the major monoterpene of eucalyptus oil, as it can represent between 60 and 80% of the volatile oils derived from eucalyptus leaves depending on the species. Therapeutic concentrations of 1,8-cineol (1.5 *µ*g/mL = 10^−5^M) inhibited significantly cytokine production in lymphocytes and monocytes [47, 48]. It has to be noted that 1,8-cineol has already gained market acceptance for its anti-inflammatory properties in mouthwashes and cough suppressants or antiasthmatic medications [48, 49].

The plants studied here were chosen on the basis of their traditional use to treat inflammatory conditions by the Dharawal people of the Campbelltown region (Southwest Sydney Australia). All of the plants showed anti-inflammatory activity and demonstrated inhibitory effect on downregulation of NO and TNF-*α* production with varying potencies, which supports their use in traditional Aboriginal medicine. The content of the anti-inflammatory compounds in the plants, according to traditional knowledge, is also dependent on the plant's environment. In Dharawal country, what is most important when seeking particular medicines from plants is where the plant is growing, that is, not so much the soils, but the other plants that are growing around the particular plant required. For instance, with the Eucalypts, close proximity of an Ironbark (Muggago) and a Ribbon bark (Kai'yeroo) is needed for the anti-inflammatory medicine from the Burringoa (*Eucalyptus tereticornis*) to be most effective. As another example, the Ironbark itself does not need other Eucalypts close by, but it does need the Einadia (one of the saltbushes) to be growing at its base. In addition, if it had been struck by lightning (and this can be confirmed by a line of interrupted bark running from the top of the tree almost to its base), then the anti-inflammatory medicine would be most effective, when using the leaves of the Eucalypts as medicine, the leaves of the trees younger than 7 years were placed on a low fire and the smoke inhaled. However, when the tree is bearing the mature leaves, the leaves were collected and boiled then allowed to cool before being rubbed on the affected part of the body, depending on the species. For the present screening study, the plant material was provided by the Botanical Gardens from random trees in the garden, but for future studies, we will investigate if collection practice based on Dharawal knowledge will improve the inherent activity and/or yield of the anti-inflammatory compounds.

## 4. Conclusions

The present study suggests that most of the* Eucalyptus* spp. potentially possess interesting anti-inflammatory compounds with low toxicity and the in vitro activity appears to support the traditional use.* Eucalyptus benthamii, E. bosistoana, E. botryoides, E. saligna, E. smithii, E. umbra, and E. viminalis* leaf extracts exhibited strong anti-inflammatory activity by inhibiting NO and TNF-*α* production in LPS and INF-*γ* stimulated RAW 264.7 macrophages. Purification and structure identification of the most these extracts are currently underway.

## Supplementary Material

Dose-response curves showing the anti-inflammatory activity of the plant extracts.

## Figures and Tables

**Table 1:** Plants collected for the study of anti-inflammatory activity.

Number	Plant	APNI name	Family	Voucher number
(1)	*Eucalyptus acmenoides*	*Eucalyptus acmenoides *Schauer	Myrtaceae	961604
(2)	*Eucalyptus benthamii*	*Eucalyptus benthamii* Maiden & Cambage	Myrtaceae	832452
(3)	*Eucalyptus bosistoana*	*Eucalyptus bosistoana* F. Muell.	Myrtaceae	20070782
(4)	*Eucalyptus botryoides*	*Eucalyptus botryoides* Sm.	Myrtaceae	861776
(5)	*Eucalyptus eximia*	*Eucalyptus eximia* Schauer	Myrtaceae	841857
(6)	*Eucalyptus globoidea*	*Eucalyptus globoidea* Blakely	Myrtaceae	873240
(7)	*Eucalyptus gummifera*	*Eucalyptus gummifera* (Gaertn.) Hochr.	Myrtaceae	892074
(8)	*Eucalyptus maculata*	*Eucalyptus maculata* Hook.	Myrtaceae	20070782
(9)	*Eucalyptus notabilis*	*Eucalyptus notabilis* Maiden	Myrtaceae	20020217
(10)	*Eucalyptus paniculata*	*Eucalyptus paniculata* Sm.	Myrtaceae	840775
(11)	*Eucalyptus pilularis*	*Eucalyptus pilularis* Sm.	Myrtaceae	861796
(12)	*Eucalyptus punctata*	*Eucalyptus punctata* DC.	Myrtaceae	861820
(13)	*Eucalyptus resinifera*	*Eucalyptus resinifera* Sm.	Myrtaceae	911862
(14)	*Eucalyptus saligna*	*Eucalyptus saligna* Sm.	Myrtaceae	872719
(15)	*Eucalyptus smithii*	*Eucalyptus smithii* R. T. Baker	Myrtaceae	361827
(16)	*Eucalyptus umbra*	*Eucalyptus umbra* R. T. Baker	Myrtaceae	900782
(17)	*Eucalyptus viminalis*	*Eucalyptus viminalis* Labill.	Myrtaceae	861830

**Table 2:** Plant common names, ethnomedicine, and yields of ethanolic extracts for the study of anti-inflammatory activity.

Plant species	Common name(s)	Diseases treated using leaves (according to Dharawal Aboriginal medicinal use)	Yield of ethanol extract (%)
*Eucalyptus acmenoides*	White mahogany/yellow stringybark	Breathing difficulties, chest and muscle pain, fever, and wash for joints	19.2
*Eucalyptus benthamii*	Camden white gum	Colds, fever, chest and muscle pain, and wash for joints	12.6
*Eucalyptus bosistoana*	Coastal grey box	Colds, fever, chest and muscle pain, and wash for joints	11.6
*Eucalyptus botryoides*	Bangalay/southern mahogany	Colds, fever, chest and muscle pain, and wash for joints	41.6
*Eucalyptus eximia*	Yellow bloodwood	Colds, fever, chest and muscle pain, wash for joints, extreme diarrhea, and syphilitic sores	25.8
*Eucalyptus globoidea*	White stringybark	Breathing difficulties, chest and muscle pain, fever, and wash for joints	26.2
*Eucalyptus gummifera*	Red bloodwood/bloodwood	Colds, fever, chest and muscle pain, and wash for joints	15.4
*Eucalyptus maculata*	Spotted gum	Asthma, colds, fever, chest and muscle pain, and wash for joints	12.6
*Eucalyptus notabilis*	Mountain mahogany	Colds, fever, chest and muscle pain, wash for joints, and extreme diarrhea	11.0
*Eucalyptus paniculata*	Grey ironbark	Asthma, morning sickness	16.4
*Eucalyptus pilularis*	Blackbutt	Colds, fever, chest and muscle pain, and wash for joints	23.2
*Eucalyptus punctata*	Grey gum	Breathing difficulties, stomach upset, and morning sickness	14.0
*Eucalyptus resinifera*	Red mahogany	Colds, fever, chest and muscle pain, wash for joints, and extreme diarrhea	12.0
*Eucalyptus saligna*	Sydney blue gum	Colds, fever, chest and muscle pain, and wash for joints	10.8
*Eucalyptus smithii*	Gully gum/blackbutt peppermint	Colds, fever, chest and muscle pain, and wash for joints	13.3
*Eucalyptus umbra*	Broad leafed white mahogany/white mahogany	Colds, fever, chest and muscle pain, wash for joints, and extreme diarrhea	10.5
*Eucalyptus viminalis*	Manna gum/ribbon gum/white gum	Colds, fever, chest and muscle pain, and wash for joints	19.0

**Table 3:** Anti-inflammatory activity and toxicity of extracts determined in RAW 264.7 macrophages.

Plant species	Inhibition of NO production (IC_50_ in *µ*g/mL)	Inhibition of TNF-*α* production (IC_50 _ in *µ*g/mL)	Cytotoxicity (LC_50_ in *µ*g/mL)
*Eucalyptus acmenoides*	56.93 ± 11.8	16.53 ± 5.9	296.22 ± 189.3
*Eucalyptus benthamii*	5.57 ± 1.4	2.06 ± 0.7	22.34 ± 9.3
*Eucalyptus bosistoana*	7.58 ± 1.2	8.53 ± 3.4	37.17 ± 15.6
*Eucalyptus botryoides*	16.65 ± 2.2	19.02 ± 5.4	108.40 ± 44.9
*Eucalyptus eximia*	34.14 ± 7.1	4.82 ± 1.6	64.14 ± 23.6
*Eucalyptus globoidea*	82.9 ± 12.5	50.73 ± 24.0	464.74 ± 199.7
*Eucalyptus gummifera*	108.17 ± 10.5	82.73 ± 52.3	313.45 ± 125.9
*Eucalyptus maculata*	99.94 ± 12.1	136.34 ± 78.8	110.22 ± 41.1
*Eucalyptus notabilis*	53.84 ± 7.7	27.48 ± 14.9	332.44 ± 107.5
*Eucalyptus paniculata*	130.7 ± 11.6	334.86 ± 192.7	540.46 ± 172.5
*Eucalyptus pilularis*	76.17 ± 10.3	21.09 ± 9.7	374.74 ± 190.7
*Eucalyptus punctata*	120.4 ± 15.9	115.73 ± 58.4	522.84 ± 221.4
*Eucalyptus resinifera*	81.21 ± 13.4	62.11 ± 36.0	268.59 ± 131.6
*Eucalyptus saligna*	19.77 ± 2.3	3.41 ± 1.3	101.01 ± 36.8
*Eucalyptus smithii*	17.62 ± 3.5	2.41 ± 1.1	38.96 ± 14.1
*Eucalyptus umbra*	17.69 ± 2.3	10.2 ± 4.5	236.5 ± 144.3
*Eucalyptus viminalis*	8.0 ± 1.2	16.68 ± 9.9	31.92 ± 11.9

*Note.* Results represent the mean ± SEM of 3 experiments in triplicate for NO production and cytotoxicity whereas for TNF-*α* production it is 1 experiment in triplicate.

## References

[1] Fürst R., Zündorf I. (2014). Plant-derived anti-inflammatory compounds: hopes and disappointments regarding the translation of preclinical knowledge into clinical progress. *Mediators of Inflammation*.

[2] Münch G., Schinzel R., Loske C. (1998). Alzheimer's disease—synergistic effects of glucose deficit, oxidative stress and advanced glycation endproducts. *Journal of Neural Transmission*.

[3] Retz W., Gsell W., Münch G., Rösler M., Riederer P. (1998). Free radicals in Alzheimer's disease. *Journal of Neural Transmission, Supplement*.

[4] Millington C., Sonego S., Karunaweera N. (2014). Chronic neuroinflammation in Alzheimer's disease: new perspectives on animal models and promising candidate drugs. *BioMed Research International*.

[5] Münch G., Thome J., Foley P., Schinzel R., Riederer P. (1997). Advanced glycation endproducts in ageing and Alzheimer's disease. *Brain Research Reviews*.

[6] Fuller S., Münch G., Steele M. (2009). Activated astrocytes: a therapeutic target in Alzheimer's disease?. *Expert Review of Neurotherapeutics*.

[7] Fuller S., Steele M., Münch G. (2010). Activated astroglia during chronic inflammation in Alzheimer's disease—do they neglect their neurosupportive roles?. *Mutation Research—Fundamental and Molecular Mechanisms of Mutagenesis*.

[8] Münch G., Gasic-Milenkovic J., Dukic-Stefanovic S. (2003). Microglial activation induces cell death, inhibits neurite outgrowth and causes neurite retraction of differentiated neuroblastoma cells. *Experimental Brain Research*.

[9] Kern J. K., Geier D. A., Sykes L. K., Geier M. R. (2013). Evidence of neurodegeneration in autism spectrum disorder. *Translational Neurodegeneration*.

[10] Qian L., Flood P. M., Hong J.-S. (2010). Neuroinflammation is a key player in Parkinson's disease and a prime target for therapy. *Journal of Neural Transmission*.

[11] Stuchbury G., Münch G. (2005). Alzheimer's associated inflammation, potential drug targets and future therapies. *Journal of Neural Transmission*.

[12] Venigalla M., Gyengesi E., Sharman M. J., Münch G. (2015). Novel promising therapeutics against chronic neuroinflammation and neurodegeneration in Alzheimer's disease. *Neurochemistry International*.

[13] Mrak R. E., Griffin W. S. T. (2005). Potential Inflammatory biomarkers in Alzheimer's disease. *Journal of Alzheimer's Disease*.

[14] Wong A., Lüth H.-J., Deuther-Conrad W. (2001). Advanced glycation endproducts co-localize with inducible nitric oxide synthase in Alzheimer's disease. *Brain Research*.

[15] Lüth H.-J., Münch G., Arendt T. (2002). Aberrant expression of NOS isoforms in Alzheimer's disease is structurally related to nitrotyrosine formation. *Brain Research*.

[16] Münch G., Deuther-Conrad W., Gasic-Milenkovic J. (2002). Glycoxidative stress creates a vicious cycle of neurodegeneration in Alzheimer's disease—a target for neuroprotective treatment strategies?. *Journal of Neural Transmission, Supplement*.

[17] Lüth H.-J., Ogunlade V., Kuhla B. (2005). Age- and stage-dependent accumulation of advanced glycation end products in intracellular deposits in normal and Alzheimer's disease brains. *Cerebral Cortex*.

[18] Krautwald M., Münch G. (2010). Advanced glycation end products as biomarkers and gerontotoxins—a basis to explore methylglyoxal-lowering agents for Alzheimer's disease?. *Experimental Gerontology*.

[19] Patel A., Rees S. D., Kelly M. A. (2014). Genetic variants conferring susceptibility to Alzheimer's disease in the general population; do they also predispose to dementia in Down's syndrome. *BMC Research Notes*.

[20] Heneka M. T., Golenbock D. T., Latz E. (2015). Innate immunity in Alzheimer's disease. *Nature Immunology*.

[21] Steiner N., Balez R., Karunaweera N., Lind J. M., Münch G., Ooi L. (2016). Neuroprotection of Neuro2a cells and the cytokine suppressive and anti-inflammatory mode of action of resveratrol in activated RAW264.7 macrophages and C8-B4 microglia. *Neurochemistry International*.

[22] Balez R., Steiner N., Engel M. (2016). Neuroprotective effects of apigenin against inflammation, neuronal excitability and apoptosis in an induced pluripotent stem cell model of Alzheimer’s disease. *Scientific Reports*.

[23] Venigalla M., Gyengesi E., Münch G. (2015). Curcumin and apigenin—novel and promising therapeutics against chronic neuroinflammation in Alzheimer's disease. *Neural Regeneration Research*.

[24] Williams R., Münch G., Gyengesi E., Bennett L. (2014). Bacopamonnieri (L.) exerts anti-inflammatory effects on cells of the innate immune system in vitro. *Food and Function*.

[25] Apetz N., Münch G., Govindaraghavan S., Gyengesi E. (2014). Natural compounds and plant extracts as therapeutics against chronic inflammation in Alzheimer’s disease—a translational perspective. *CNS and Neurological Disorders—Drug Targets*.

[26] Nguyen N. T. Q., Ooi L., Piller S. C., Münch G. (2013). Proenergetic effects of resveratrol in the murine neuronal cell line Neuro2a. *Molecular Nutrition & Food Research*.

[27] Raju R., Gunawardena D., Ahktar M., Low M., Reddell P., Münch G. (2016). Anti-inflammatory chemical profiling of the Australian rainforest tree Alphitonia petriei (Rhamnaceae). *Molecules*.

[28] Gunawardena D., Karunaweera N., Lee S. (2015). Anti-inflammatory activity of cinnamon (C. zeylanicum and C. cassia) extracts - Identification of E-cinnamaldehyde and o-methoxy cinnamaldehyde as the most potent bioactive compounds. *Food and Function*.

[29] Griswold D. E., Hillegass L. M., Breton J. J., Esser K. M., Adams J. L. (1993). Differentiation in vivo of classical non-steroidal antiinflammatory drugs from cytokine suppressive antiinflammatory drugs and other pharmacological classes using mouse tumour necrosis factor alpha production. *Drugs under Experimental and Clinical Research*.

[30] Denkert C., Koch I., Berger S., Köbel M., Siegert A., Hauptmann S. (2003). Cytokine-suppressive anti-inflammatory drugs (CSAIDs) inhibit invasion and MMP-1 production of ovarian carcinoma cells. *Cancer Letters*.

[31] Berbaum K., Shanmugam K., Stuchbury G., Wiede F., Körner H., Münch G. (2008). Induction of novel cytokines and chemokines by advanced glycation endproducts determined with a cytometric bead array. *Cytokine*.

[32] Gasic-Milenkovic J., Dukic-Stefanovic S., Deuther-Conrad W., Gärtner U., Münch G. (2003). *β*-amyloid peptide potentiates inflammatory responses induced by lipopolysaccharide, interferon -*γ* and 'advanced glycation endproducts' in a murine microglia cell line. *European Journal of Neuroscience*.

[33] Wong A., Dukic-Stefanovic S., Gasic-Milenkovic J. (2001). Anti-inflammatory antioxidants attenuate the expression of inducible nitric oxide synthase mediated by advanced glycation endproducts in murine microglia. *European Journal of Neuroscience*.

[34] Low M., Khoo C. S., Münch G., Govindaraghavan S., Sucher N. J. (2015). An in vitro study of anti-inflammatory activity of standardised Andrographis paniculata extracts and pure andrographolide. *BMC Complementary and Alternative Medicine*.

[35] Karunaweera N., Raju R., Gyengesi E., Munch G. (2015). Plant polyphenols as inhibitors of nf-*κ*b induced cytokine production—a potential anti-inflammatory treatment for alzheimer’s disease?. *Frontiers in Molecular Neuroscience*.

[36] Hansen E., Krautwald M., MacZurek A. E. (2010). A versatile high throughput screening system for the simultaneous identification of anti-inflammatory and neuroprotective compounds. *Journal of Alzheimer's Disease*.

[37] Dugasani S., Pichika M. R., Nadarajah V. D., Balijepalli M. K., Tandra S., Korlakunta J. N. (2010). Comparative antioxidant and anti-inflammatory effects of [6]-gingerol, [8]-gingerol, [10]-gingerol and [6]-shogaol. *Journal of Ethnopharmacology*.

[38] Zakaria Z. A., Mohamad A. S., Chear C. T., Wong Y. Y., Israf D. A., Sulaiman M. R. (2010). Antiinflammatory and antinociceptive activities of zingiber zerumbet methanol extract in experimental model systems. *Medical Principles and Practice*.

[39] Haridas V., Arntzen C. J., Gutterman J. U. (2001). Avicins, a family of triterpenoid saponins from Acacia victoriae (Bentham), inhibit activation of nuclear factor-*κ*B by inhibiting both its nuclear localization and ability to bind DNA. *Proceedings of the National Academy of Sciences of the United States of America*.

[40] Li R. W., Leach D. N., Myers S. P. (2004). Anti-inflammatory activity, cytotoxicity and active compounds of Tinospora smilacina Benth. *Phytotherapy Research*.

[41] Tan A. C., Konczak I., Ramzan I., Zabaras D., Sze D. M.-Y. (2011). Potential antioxidant, antiinflammatory, and proapoptotic anticancer activities of Kakadu plum and Illawarra plum polyphenolic fractions. *Nutrition and Cancer*.

[42] Sweeney A. P., Wyllie S. G., Shalliker R. A., Markham J. L. (2001). Xanthine oxidase inhibitory activity of selected Australian native plants. *Journal of Ethnopharmacology*.

[43] Byard R. (1988). Traditional medicine of aboriginal Australia. *Canadian Medical Association Journal*.

[44] Hart P. H., Brand C., Carson C. F., Riley T. V., Prager R. H., Finlay-Jones J. J. (2000). Terpinen-4-ol, the main component of the essential oil of Melaleuca alternifolia (tea tree oil), suppresses inflammatory mediator production by activated human monocytes. *Inflammation Research*.

[45] Juergens U. R. (2014). Anti-inflammatory properties of the monoterpene 18-cineole: current evidence for co-medication in inflammatory airway diseases. *Drug Research*.

[46] O'Brien J., Wilson I., Orton T., Pognan F. (2000). Investigation of the Alamar Blue (resazurin) fluorescent dye for the assessment of mammalian cell cytotoxicity. *European Journal of Biochemistry*.

[47] Juergens U. R., Stöber M., Vetter H. (1998). Inhibition of cytokine production and arachidonic acid metabolism by eucalyptol (1.8-cineole) in human blood monocytes in vitro. *European Journal of Medical Research*.

[48] Juergens U. R., Dethlefsen U., Steinkamp G., Gillissen A., Repges R., Vetter H. (2003). Anti-inflammatory activity of 1.8-cineol (eucalyptol) in bronchial asthma: a double-blind placebo-controlled trial. *Respiratory Medicine*.

[49] Yu R., Mandlekar S., Lei W., Fahl W. E., Tan T.-H., Kong A.-N. T. (2000). p38 Mitogen-activated protein kinase negatively regulates the induction of phase II drug-metabolizing enzymes that detoxify carcinogens. *The Journal of Biological Chemistry*.

