# Supplementary material for: Medicinal Plants of the Australian Aboriginal Dharawal People Exhibiting Anti-Inflammatory Activity
Source: Evid Based Complement Alternat Med. 2016 Dec 27;2016:2935403. doi: 10.1155/2016/2935403 (PMC5223016; doi:10.1155/2016/2935403)
Supplement: Supplementary file 1 — Dose-response curves showing the anti-inflammatory activity of the plant extracts. [file 2935403.f1.pdf]

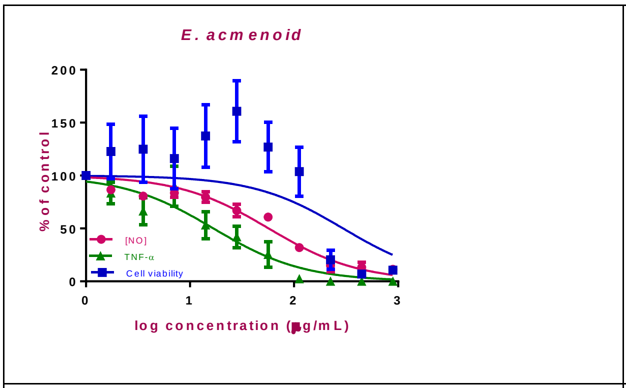
E. acmenoid
% of control
log concentration (µg/mL)
[NO]
TNF-α
Cell viability

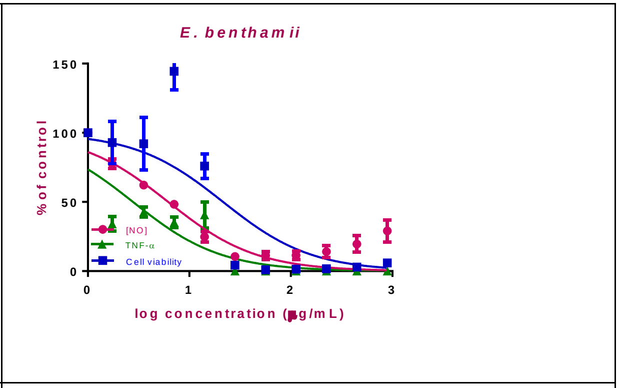
E. benthamii
% of control
log concentration (µg/mL)
[NO]
TNF-α
Cell viability

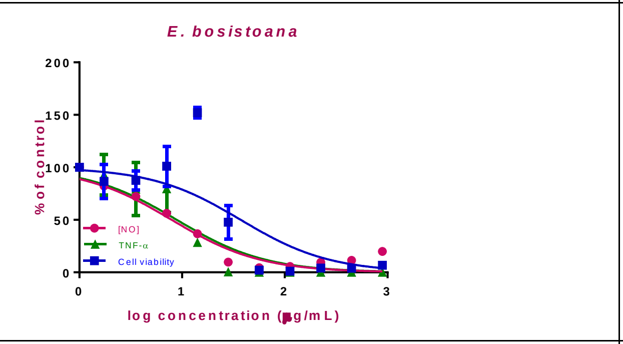
E. bosistoana
% of control
log concentration (µg/mL)
[NO]
TNF-α
Cell viability

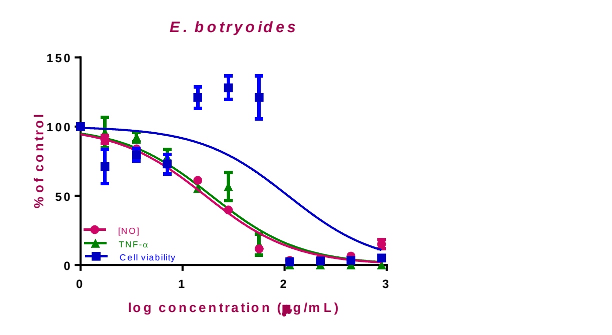
E. botryoides
% of control
log concentration (µg/mL)
[NO]
TNF-α
Cell viability

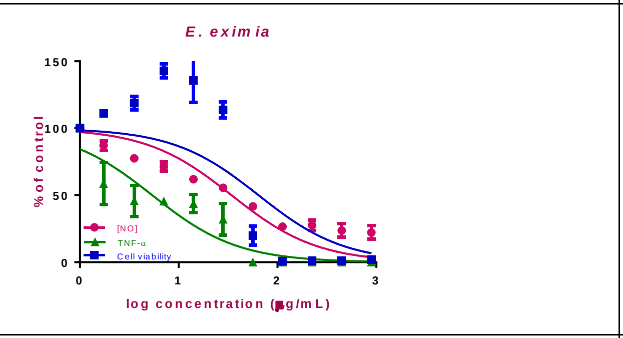
E. eximia
% of control
log concentration (µg/mL)
[NO]
TNF-α
Cell viability

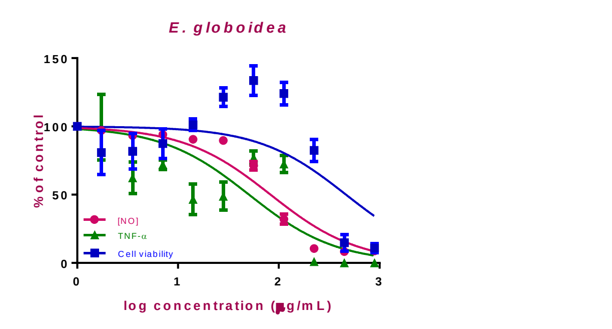
E. globoidea
% of control
log concentration (µg/mL)
[NO]
TNF-α
Cell viability

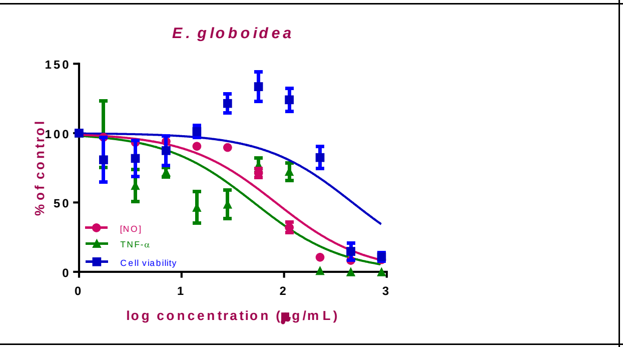
E. globoidea
% of control
log concentration (µg/mL)
[NO]
TNF-α
Cell viability

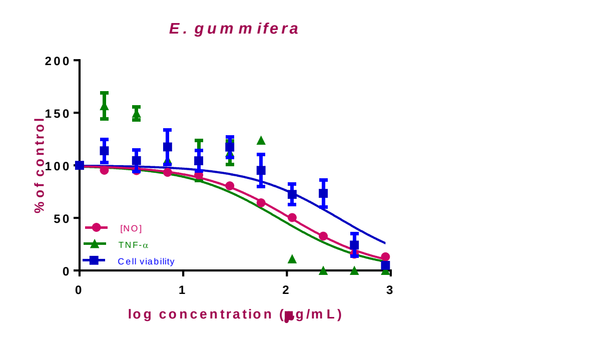
E. gummifera
% of control
log concentration (µg/mL)
[NO]
TNF-α
Cell viability

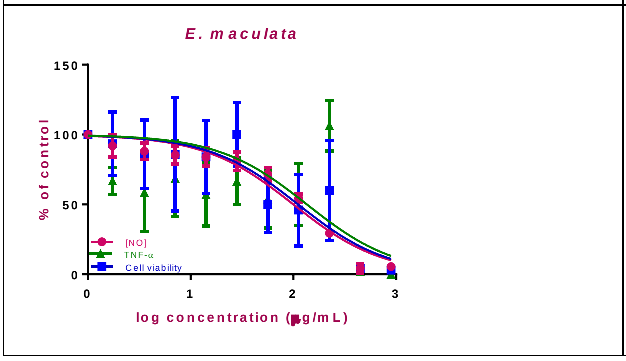
E. maculata
% of control
log concentration (µg/mL)
[NO]
TNF-α
Cell viability

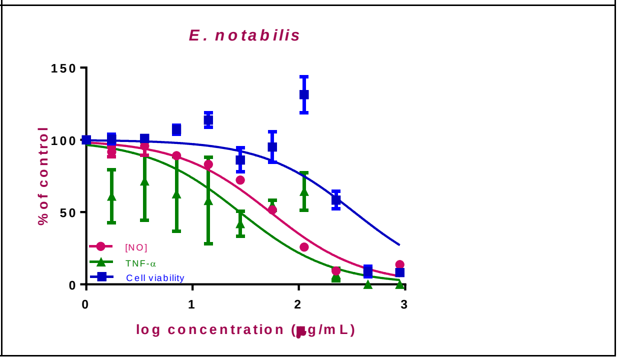
E. notabilis
% of control
log concentration (µg/mL)
[NO]
TNF-α
Cell viability

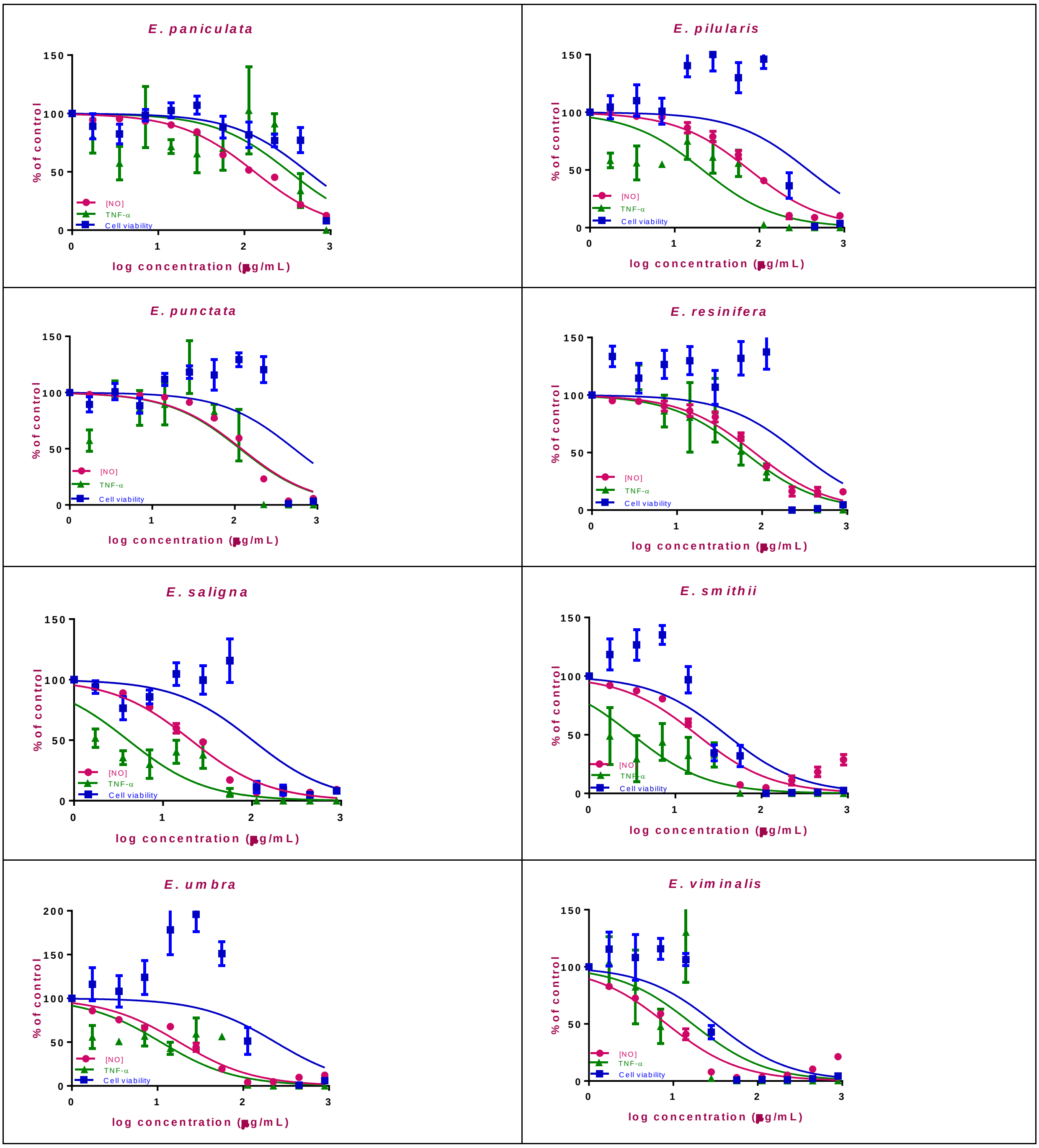

Supplementary figure 1: Inhibition of LPS and IFN-γ induced production of pro-inflammatory markers by ethanolic extracts from seventeen *Eucalyptus* species.

Results are represented as mean ± SEM of 3 experiments (in triplicate) for nitric oxide production and cell viability and one experiment (in triplicate) for TNF-α production.
